# Targeted *SMN* Exon Skipping: A Useful Control to Assess In Vitro and In Vivo Splice-Switching Studies

**DOI:** 10.3390/biomedicines9050552

**Published:** 2021-05-14

**Authors:** Loren L. Flynn, Chalermchai Mitrpant, Abbie Adams, Ianthe L. Pitout, Anja Stirnweiss, Sue Fletcher, Steve D. Wilton

**Affiliations:** 1 Centre for Molecular Medicine and Innovative Therapeutics, Health Futures Institute, Murdoch University, Murdoch, WA 6150, Australia; loren.flynn@murdoch.edu.au (L.L.F.); a.adams@murdoch.edu.au (A.A.); i.pitout@murdoch.edu.au (I.L.P.); S.fletcher@murdoch.edu.au (S.F.); 2 Perron Institute for Neurological and Translational Science, Nedlands, WA 6009, Australia; chalermchai.mit@mahidol.edu; 3 Centre for Neuromuscular & Neurological Disorders, University of Western Australia, Crawley, WA 6009, Australia; 4 Black Swan Pharmaceuticals, Wake Forest, NC 27587, USA; 5 Department of Biochemistry, Faculty of Medicine Siriraj Hospital, Mahidol University, Bangkok 10700, Thailand; 6 PYC Therapeutics, Nedlands, WA 6009, Australia; anja.stirnweiss@pyctx.com

**Keywords:** antisense oligonucleotide, morpholino, positive control, survival motor neuron, cell penetrating peptide

## Abstract

The literature surrounding the use of antisense oligonucleotides continues to grow, with new disease and mechanistic applications constantly evolving. Furthermore, the discovery and advancement of novel chemistries continues to improve antisense delivery, stability and effectiveness. For each new application, a rational sequence design is recommended for each oligomer, as is chemistry and delivery optimization. To confirm oligomer delivery and antisense activity, a positive control AO sequence with well characterized target-specific effects is recommended. Here, we describe splice-switching antisense oligomer sequences targeting the ubiquitously expressed human and mouse *SMN* and *Smn* genes for use as control AOs for this purpose. We report two AO sequences that induce targeted skipping of SMN1/SMN2 exon 7 and two sequences targeting the *Smn* gene, that induce skipping of exon 5 and exon 7. These antisense sequences proved effective in inducing alternative splicing in both in vitro and in vivo models and are therefore broadly applicable as controls. Not surprisingly, we discovered a number of differences in efficiency of exon removal between the two species, further highlighting the differences in splice regulation between species.

## 1. Introduction

Antisense oligonucleotides (AOs) are synthetic nucleic acid analogues, specifically designed to regulate target gene transcripts and protein expression. Common mechanistic strategies include: RNase H-mediated transcript degradation [1]; preventing the initiation of protein translation [2]; and modulating pre-mRNA splicing to either produce alternative functional protein isoforms, to overcome mutations or errors in splicing, or to produce a non-functional transcript [3,4,5,6]. The application of splice-switching antisense therapies to treat a broad variety of genetic disorders has greatly expanded over the past decade, with antisense drugs to treat Duchenne muscular dystrophy and spinal muscular atrophy now available in the clinic. For a review of the translational development of splice-switching antisense therapies, see Pitout et al, 2019 [7].

We have previously reported our recommendations for the efficient and rational design of AO sequences for splice-modulation, as well as the optimization of delivery and choice of AO chemistry for improved splice-switching effects [8,9]. In particular, AO delivery is of paramount importance and hence should be optimized through the comparison of multiple transfection reagents and delivery techniques when a new gene transcript or cell line is investigated. Traditionally, the use of a fluorescently tagged AO has been recommended for optimization of transfection efficiency [10,11]; however, the addition of a fluorescent tag or similar conjugate increases the molecular weight of the AO and likely influences the uptake of the compound, while cleavage of the tag within the cytoplasm may confound interpretation of AO localization. Visualization of a fluorescently labelled AO is limited in identifying that the AO permeates the cell but does not confirm AO escape from the endosome, which is necessary for biological availability and activity of the AO [12,13]. Furthermore, fluorescent AOs are unsuitable for in vivo applications. An antisense sequence targeting a ubiquitously expressed and efficiently modified transcript is, therefore, an ideal compound for transfection optimization and for use as a positive control in subsequent splice manipulation experiments. The effects of such a splice-switching control AO can allow quantitation of transfection efficiency and can characterize optimal transfection parameters. Importantly, the use of a positive control AO targeting a ubiquitously expressed gene serves as an ideal positive control for broad tissue targeting and in vivo evaluation of new AO chemistries and modifications.

We have extensive experience with developing AO sequences for exon skipping and routinely screen 2′-*O*-methyl phosphorothioate (PS) AOs and phosphorodiamidate morpholinos (PMOs) to identify lead drug candidates. While 2′-*O*-methyl PS AOs are suitable for in vitro screening to identify the optimal target binding site, off-target binding and adverse events due to the negatively charged phosphorothioate backbone make them less suitable for in vivo and clinical applications [14,15,16]. Conversely, PMOs have an excellent safety profile, and when efficiently delivered, are more effective than their 2′-*O*-methyl counterparts when studying protein changes or functional assays [17]. Delivery of PMOs in vivo typically requires high dosing and/or frequent administration [18]. Therefore, efforts to enhance delivery have focused on conjugating PMOs to cell penetrating peptides (PPMOs), improving cellular uptake by up to 10-fold [18,19,20,21]. We have developed a robust positive control AO targeting the *SMN/Smn* transcripts that can be used to optimize and evaluate delivery of antisense nucleic acid analogues and identify tissue-specific or differential delivery in vivo.

**Figure 1:**
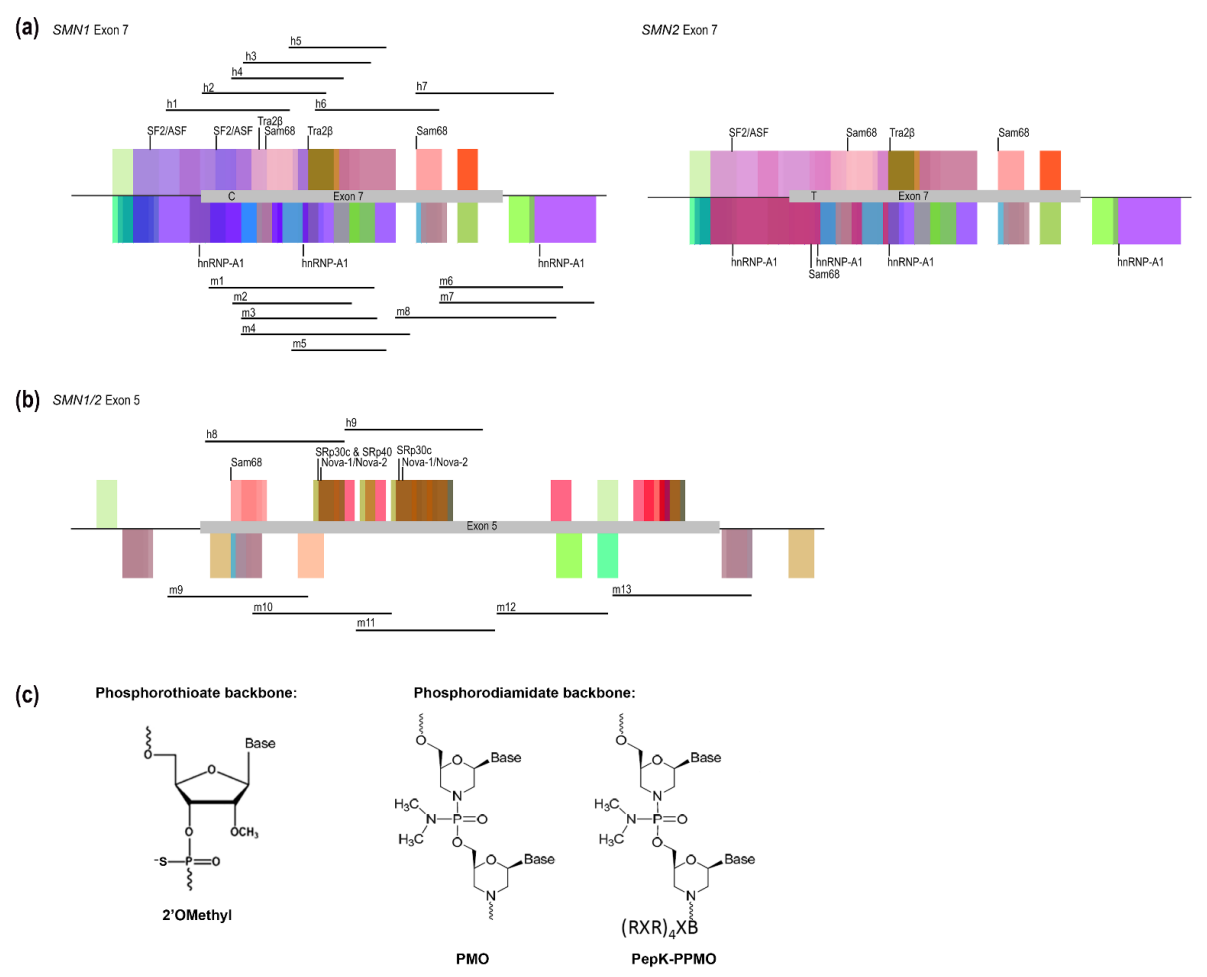
(**a**) schematic of *SMN1* and *SMN2* exon 7 SpliceAid predicted splicing factor binding sites with the more strongly predicted splicing factors labelled, including alternative splicing factor/pre-mRNA splicing factor 2 (ASF/SF2) and transformer 2 Beta (Tra2β) as positive exonic splicing enhancer elements, and SRC associated in mitosis of 68 kDa (Sam68) and heterogenous nuclear ribonucleoprotein A1 (hnRNP-A1) as negative exonic splicing silencer elements. The binding location of the human exon 7 skipping AOs are indicated above the *SMN1* exon 7, and the homologous mouse *Smn* exon 7 skipping AOs are indicated below; (**b**) schematic of *SMN1/2* exon 5 predicted splicing factor binding sites, with the more strongly predicted splicing factors labelled, including Sam68, serine and arginine rich splicing factors (SRp-30c and SRp-40) and the Nova-1 and Nova-2 proteins as exonic splicing enhancers. The human and mouse exon 5 skipping AOs are indicated above and below the schematic, respectively; and (**c**) the chemical structure of the three AO chemistries used in this study, including the 2′-*O*-methyl phosphorothioate AO, the PMO and the PepK-conjugated PPMO, adapted from [27,28].

The survival motor neuron (SMN) protein is ubiquitously expressed, and its reduced expression is associated with the childhood disease spinal muscular atrophy (SMA) due to the functional loss of the *SMN1* gene. A single base change in exon 7 between the two genes encoding SMN, *SMN1* and *SMN2* modifies splicing factor binding, leading to the alternative splicing of that in-frame exon (Figure 1a). While AOs targeting an intronic silencer element downstream of *SMN2* exon 7 have been developed to increase SMN levels to overcome SMA [22,23,24], AOs inducing skipping of exon 7 from the *SMN1* transcript can be used to induce and model disease [25], as well as act as positive controls for separate AO studies. Here, we report sequences targeting in-frame exons 5 and 7 of the *SMN1/SMN2* and *Smn* transcripts that can be used as antisense controls for optimizing AO delivery and chemistry in both in vitro and in vivo models. Not surprisingly, we again observed substantial variation in the efficiencies of exons 5 and 7 removal, between human and mouse transcripts, highlighting differences in splice-regulation between species [26].

## 2. Materials and Methods

### 2.1. AO Design

AOs were initially designed to induce skipping of exon 7 from the human and mouse *SMN/Smn* genes, and following these results, to induce exon 5 skipping in both species. Human-specific AOs were designed to target exonic splicing enhancer (ESE) domains as predicted by the online SpliceAid prediction tool, available at http://www.introni.it/splicing.html, last accessed on 9 May 2021 [29]. Mouse-specific AOs were designed to target ESEs as predicted by the online Rescue-ESE program, available at http://genes.mit.edu/burgelab/rescue-ese/, last accessed on 19 December 2014 [30]. The predicted splicing factor binding sites and the AO target sites for both the human and mouse exons are illustrated in Figure 1a,b respectively. AO nomenclature was based on that described by Mann et al. (2002) [31]. All 2′-*O*-methyl AOs were synthesized in house on an Expedite 8909 nucleic acid synthesizer with a phosphorothioate (PS) backbone. All AOs tested in this study, their sequences and binding coordinates are listed in Table 1. The chemical structure for each of the AO chemistries utilized in this study, the 2′-*O*-methyl phosphorothioate, the phosphorodiamidate morpholino (PMO) and the PepK-conjugated PMO are illustrated in Figure 1c.

### 2.2. Cell Plating and 2′-O-Methyl Phosphorothioate AO Transfection

Normal human dermal fibroblasts prepared in-house (Murdoch University Human Research Ethics Committee Approval #2013/156) were proliferated and seeded (7500 cells/cm^2^) in 10% FBS DMEM and incubated overnight prior to 2′-*O*-methyl PS-AO transfection. Transfections were performed using Lipofectamine 3000 (Life Technologies, Melbourne, Australia) with 3 µL of Lipofectamine 3000 per 1 mL of transfection volume, according to the manufacturer’s protocol, and incubated for 48 h. AO-lipid complexes were made up in OptiMEM (Life Technologies).

Primary mouse *mdx* myoblasts were seeded onto Matrigel (Becton Dickinson Life Technologies) pre-coated plates (100 µg/mL, 37 °C for 1 h) in 20% FBS, 0.5% chick embryo extract, Ham’s-F10 and incubated overnight prior to transfection. 2′-*O*-methyl PS-AOs were transfected into mouse cells using Lipofectin (Life Technologies, Melbourne, Australia) at a 2:1 ratio of Lipofectin to total AO, in OptiMEM, according to manufacturer’s protocols, and incubated for 48 h.

### 2.3. Culture and Differentiation of SH-SY5Y Cells

The SH-SY5Y neuroblastoma cell line was maintained in 10% FBS EMEM/Hams-F12 (1:1). For differentiation into neuronal-like cells, SH-SHY5Y cells were plated onto Matrigel pre-coated plates (100 µL/cm^2^, 37 °C for 1 h) and incubated for 7 days in 1% FBS EMEM/Hams-F12 with 10 µM Retinoic Acid (Sigma-Aldrich, Sydney, Australia). The media was replaced every two days.

### 2.4. Transfection of Phosphorodiamidate Morpholino Oligomers and Peptide Conjugated PMOs

Human and mouse PMOs were purchased from Gene Tools LLC (Philomath, OR, U.S.A.) The human PepK-conjugated PPMO was provided by Sarepta Therapeutics (Cambridge, MA, U.S.A.), and the mouse PepK-conjugated PPMO was synthesised by Cambridge Research Biochemicals (Cleveland, UK) and provided by PYC Therapeutics (Perth, Australia). The PepK chemical composition can be found in [32] and the PPMO structure illustrated in Figure 1c. PMOs and PPMOs were transfected un-complexed at doses ranging from 100 nM to 10 µM. P/PMOs were diluted accordingly in OptiMEM and applied directly to the cells. Transfected cells were incubated at 37 °C for 3 days prior to cell harvesting.

### 2.5. RNA Extraction, Polymerase Chain Reaction, Visualisation and Analysis

RNA was extracted using the MagMAX-96 Total RNA Isolation Kit, including a DNase treatment (Life Technologies), according to the manufacturer’s instructions. RT-PCRs were performed using the One-step Superscript III RT-PCR kit with Platinum Taq polymerase (Life Technologies) according to the manufacturer’s instructions. All primer sequences and PCR conditions used in this study are detailed in Table 2. All PCR products were separated on a 2% agarose gel, stained with RedSafe (iNtRON Biotechnology, Korea) and visualized on a Vilber Lourmat Fusion FX System (Germany). Bio-1D Software (Vilber Lourmat) was used for densitometry analysis.

### 2.6. In Silico Analysis of SMN and Smn Splice Site Scores

In silico analysis of the human and mouse exon 5 and 7 splice sites was preformed using the MaxEntScan (MES) prediction tool (available online: http://hollywood.mit.edu/burgelab/maxent/Xmaxentscan_scoreseq_acc.html, last accessed 19 February 2021) [33] to determine the predictive strength of the respective splice sites, based on the maximum entropy principle.

### 2.7. In Vivo Evaluation of a Peptide-Conjugated PMO

C57BL/10ScSnDmd^mdx^ (mdx) mice were supplied by the Animal Resources Centre (Murdoch, Australia) and housed at the Small Animal Facility, Murdoch University according to the National Health and Medical Research Council Code of Practice. All experiments performed on animals were approved by the Murdoch University Animal Ethics Committee (approval number R2625/13). As a proof of concept experiment and to determine dosage for future experiments, an *mdx* pup (postnatal day 5 (P5)) was injected with a single subcutaneous injection (2 nmol) and a single intraperitoneal injection (2 nmol) (combined oligomer dosage of 4 nmol) of PepK peptide-conjugated PMO in normal saline. The Smn-PMO and the PepK peptide alone were used as delivery controls, whereby *mdx* mice were treated with 3 subcutaneous injections (2 nmol) on P5, P7 and P9 (combined PMO or peptide dosage of 6 nmol). Treated mice were sacrificed 7 days following the initial injection and tissue sampled and cryopreserved for *Smn* detection. Tissues were sectioned on a cryostat and lysed for RNA extraction and RT-PCR analysis as above.

## 3. Results

### 3.1. Screening of 2′-O-Methyl AOs Targeting SMN Exons 5 and 7 in Normal Human Fibroblasts

**Figure 2:**
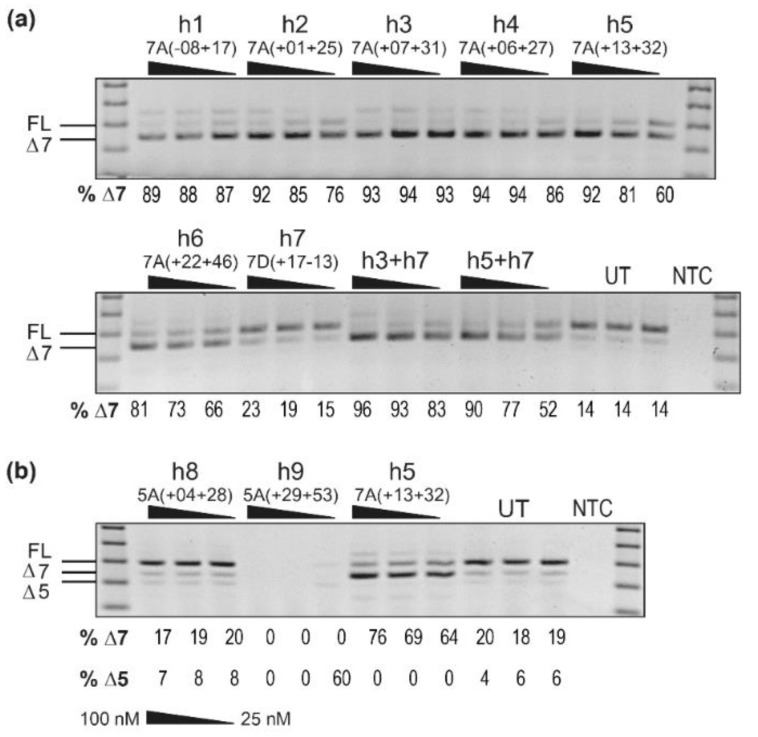
RT-PCR analysis of *SMN* transcripts across exons 4–8 in normal human fibroblasts transfected with 2′-*O*-methyl PS AOs (100, 50 and 25 nM). The full length transcript (FL, 404 bp) and exon skipped products (delta-7(Δ7), 350 bp and delta-5(Δ5), 308 bp) are indicated by the lines to the left of the gel, and the percentage of (**a**) Δ7-*SMN* or (**b**) Δ7-*SMN* and Δ5-*SMN* as determined by densitometry is shown below each lane for comparison to controls and untreated (UT) fibroblasts. A 100 bp size marker was used for size comparison and a no template RT-PCR control (NTC) was loaded in the final lane.

Antisense oligomers composed of 2′-*O*-methyl modified bases on a phosphorothioate backbone were designed to target acceptor and donor splice sites and ESE domains across exons 5 and 7 of the *SMN1*/*SMN2* genes. Initially, AOs designed to induce *SMN* exon 7 skipping were transfected into normal human fibroblasts using Lipofectamine 3000 (Life Technologies, Melbourne, Australia) at 100, 50 and 25 nM. RT-PCR analysis and gel fractionation (Figure 2) revealed a larger product above the expected full-length amplicon, consistent with a PCR heteroduplex. Antisense oligomers designed to target exon 7 were found be effective at inducing exon 7 skipping, with a clear dose response induced by all AOs tested. Sequences targeting the acceptor splice site and internal ESEs appear to be the most effective, with up to 90% exon skipping induced by the majority of AOs at 100 nM, while an AO targeting the donor splice site was clearly less efficient with only 23% exon 7 skipping in cells after transfection at the higher concentration. AOs h3 (25mer), h4 (22mer) and h5 (20mer) all induced the highest levels of exon skipping at 100 nM, and therefore, these sequences could be interchangeable as positive controls if AOs of different lengths were needed. However, consistent with the principle of using the shorter AOs when possible, h5 was selected for future studies. When comparing the sequences reported here with those reported by others [25], there was no notable difference in exon skipping effect (not shown).

To ascertain whether exon skipping could be enhanced by non-overlapping AOs in combination, two cocktails were tested, combining the h7 donor site AO with effective AOs targeting the middle of the exon, h3 (H7A(+ 07 + 31)), and h5 (H7A(+ 13 + 32)) at a 1:1 ratio. These cocktails induced lower levels of exon 7 skipping compared to the h3 and h5 AOs alone.

To compare AO-mediated exon 5 and 7 skipping from the human *SMN* transcripts, two exon 5 skipping AOs were tested in normal human fibroblasts, for comparison to exon 7 skipping (Figure 2b). Analysis of predicted exon 5 and 7 splice site maximum entropy scores using MaxEntScan [33] (Table 3) revealed exon 5 to be the “weaker” exon with an acceptor site MES score of 5.44, compared to exon 7 with an acceptor score of 10.92. Contrary to this observation, and although removal of exon 5 can still produce a semi-functional transcript [34], AO-induced exon 5 skipping in the human transcript was less efficient than exon 7 skipping. AO h8 (SMN H5A(+ 04 + 28)) was not effective, with only 7% of transcripts showing skipping of exon 5, while AO h9 targeting SMN H5A(+ 29 + 53) appears to completely knockdown full length *SMN* transcript levels.

### 3.2. In Silico Analysis of Human and Mouse Exon Splice Site Scores

In silico analysis of the human and mouse exon 5 and 7 splice sites was preformed using the MaxEntScan (MES) prediction tool (available at http://hollywood.mit.edu/burgelab/maxent/Xmaxentscan_scoreseq_acc.html, accessed on 19 February 2021) [33] to determine the predictive strength of the splice sites, based on the maximum entropy principle (Table 3). Within both the human and mouse gene transcripts, the exon 5 acceptor site had low predicted MES scores, 5.44 and 3.29, respectively, out of a possible 12, suggesting that this is a weak splice site in both species, particularly so in the mouse. While there was no obvious difference in the MES scores for the exon 7 acceptor sites across the species, the mouse exon 7 donor site was predicted to be slightly stronger (9.82) when compared to the human splice motif (8.57); however, this 13% difference in MES score did not meet the 15% threshold predicted to alter splicing outcomes [35].

### 3.3. Using the SMN Exon Skipping AO as a Positive Control for Optimising AO Chemistry and Delivery Across Cell Lines

Following identification of the lead human *SMN* exon skipping sequence, the h5 sequence was used as a positive control to optimize and compare AO chemistries and delivery into different cell types. The h5 sequence targeting H7A(+ 13 + 32) was synthesized as a more clinically applicable PMO antisense chemistry (SMN-PMO), due to its excellent safety profile. To enhance PMO delivery, we compared the PMO alone and when conjugated to PepK, an arginine-rich cell penetrating peptide (SMN-PPMO), to enhance cellular uptake and exon skipping effect [32].

**Figure 3:**
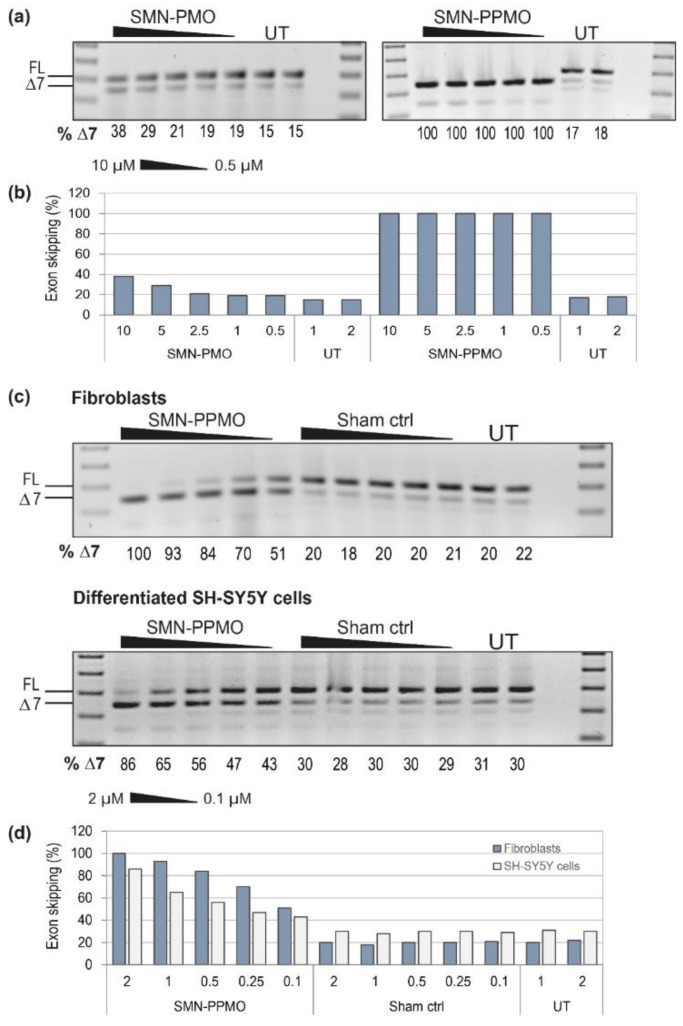
Analysis of AO chemistry and delivery after transfection into fibroblasts and neuronal cells, showing (**a**) RT-PCR across *SMN* transcripts following transfection with exon skipping PMO and PPMO into fibroblasts at 10, 5, 2.5, 1 and 0.5 µM; (**b**) graphical representation of densitometry as in (**a**); (**c**) RT-PCR of *SMN* transcripts in fibroblasts and SH-SY5Y cells, following transfection with the SMN-PPMO at 2, 1, 0.5, 0.25 and 0.1 µM; and (**d**) graphical representation of densitometry as in (**c**). The full length transcript (FL, 404 bp) and exon 7 skipped product (delta-7(Δ7), 350 bp) are indicated by the lines to the left of the gel, and the percentage of Δ7-*SMN* transcript, as determined by densitometry is shown below each lane for comparison to controls and untreated (UT) cells. A 100 bp size marker was used for size comparison and a no template RT-PCR control (NTC) was loaded in the final lane.

The SMN-PMO and PPMO were evaluated in human fibroblasts, transfected without a delivery agent at 10, 5, 2.5, 1 and 0.5 µM, and the transfected cells incubated for 3 days. RT-PCR analysis and gel fractionation of *SMN* transcripts (Figure 3a) revealed the PMO to be inefficient when delivered uncomplexed, with a maximum of 38% of transcripts missing exon 7 after transfection at the higher concentration, compared to 15% in untreated cells. In comparison, the PepK-conjugated PPMO was particularly effective, with 100% exon 7 skipping induced at all concentrations tested. The presence of a smaller 254 bp PCR product was confirmed to be the *SMN* transcript missing both exons 5 and 7, and endogenous Δ5-*SMN* was also detected in untreated samples from these cells.

The SMN-PPMO was used as a positive control to optimize transfection concentration and PMO delivery into neuronal cells, compared to fibroblasts. For neuronal evaluation, the SH-SY5Y neuroblastoma cell line was differentiated into neurons using 10 µM retinoic acid. Fibroblasts and differentiated SH-SY5Y cells were then transfected with SMN-PPMO at 2, 1, 0.5, 0.25 and 0.1 µM, and incubated for 3 days. RT-PCR analysis of *SMN* transcripts (Figure 3c) shows 86% Δ7-*SMN* transcript in cells transfected with the SMN-PPMO at 2 µM, with a clear dose response. Interestingly, exon 7 skipping was less efficient in the neurons compared to primary fibroblasts, whereby 100% skipping was observed in cells transfected with the SMN-PPMO at a concentration of 2 µM.

### 3.4. Screening of 2′-O-Methyl AOs Targeting Smn Exon 7 in Mdx Myoblasts

**Figure 4:**
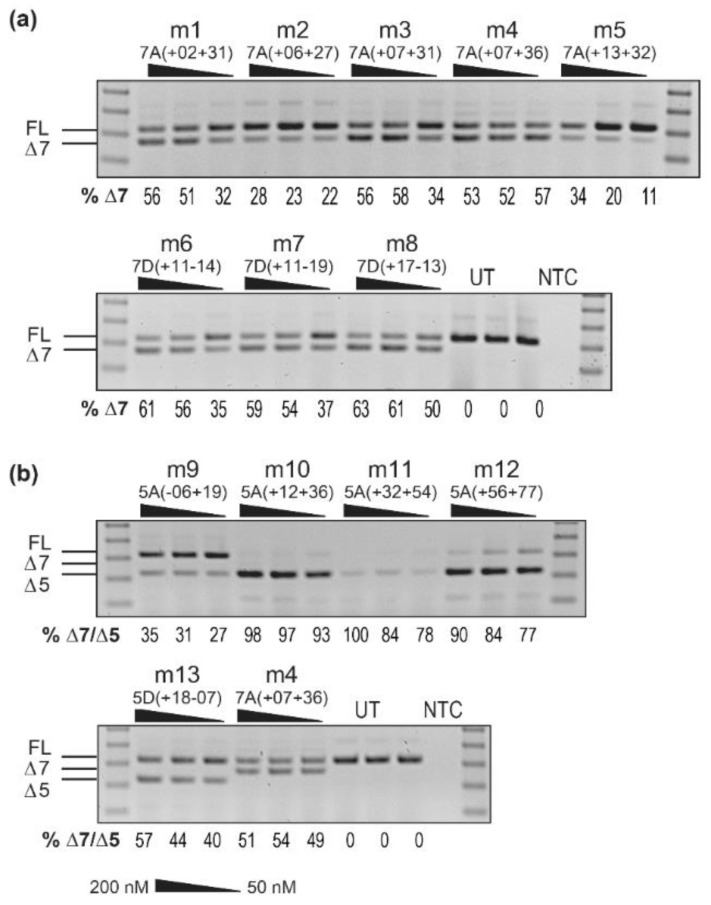
RT-PCR analysis of *Smn* transcripts across exons 4 to 8 in *mdx* mouse myoblasts. Myoblasts were transfected with individual 2′-*O*-methyl PS AOs (200, 100 and 50 nM), targeting (**a**) exon 7, and (**b**) exons 5 and 7. The full length transcript (FL, 430 bp) and exon skipped products (delta-7(Δ7), 380 bp and delta-5(Δ5), 334 bp) are indicated by the lines to the left of the gel. The percentage of Δ7-*Smn* and Δ5-*Smn* transcripts, as determined by densitometry, are shown below each lane for comparison to controls of untreated (UT) myoblasts. A 100 bp size marker was used for size comparison and a no template RT-PCR control (NTC) was loaded in the final lane.

To optimize a positive control AO targeting the mouse *Smn* sequence, 2′-*O*-methyl AOs targeting the mouse *Smn* exon 7 were transfected in *mdx* mouse myoblasts to assess exon skipping. AOs were designed to target the same co-ordinates as the human-specific AOs, and to alternative regions, depending on in silico predictions of splicing factor binding motifs. AOs were transfected into myoblasts using Lipofectamine 2000 (Life Technologies) at 200, 100 and 50 nM, with the RT-PCR result of *Smn* exon 7 skipping shown in Figure 4a. There appears to be a maximum of around 60% AO-induced exon 7 skipping from the mouse transcript, compared to over 90% AO-induced skipping from the human *SMN* transcripts. Unlike the human-specific AOs, targeting the donor splice site of the mouse exon 7 appears to cause the highest levels of skipping, with AOs m6 and m8 targeting M7D(+ 11 − 14) and M7D(+ 17 − 13) respectively, both inducing over 60% exon 7 skipping. Similar to exon 7 skipping evaluation in human cells, evaluation of AO cocktails, in an attempt to improve exon skipping, did not improve the level of *Smn* exon skipping (Supplementary Figure S1).

To compare the skipping of mouse *Smn* exons 5 and 7, 2′-*O*-methyl, AOs targeting exon 5 and exon 7 of the mouse sequence were tested at 200, 100 and 50 nM. RT-PCR results (Figure 4b) show that m4, the exon 7 30-mer targeting M7A(+ 07 + 36), induces a maximum of 50% exon 7 skipping in mouse myoblasts, while AOs targeting *Smn* exon 5 induce close to 100% Δ5-*Smn* transcripts. The sequence m10, a 25-mer targeting M5A(+ 12 + 36), induces greater than 90% skipping at 50 nM, suggesting that AOs targeting exon 5 are more effective as positive controls for mouse studies.

### 3.5. Using the Mouse Smn AO as a Positive Control for Optimizing AO Chemistry and In Vivo Delivery

**Figure 5:**
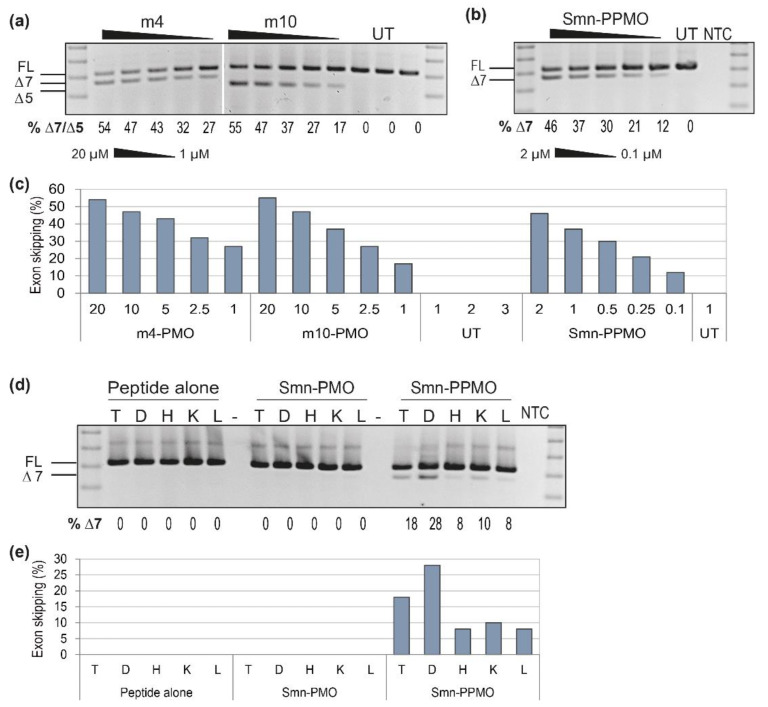
RT-PCR analysis of *Smn* products across exons 4 to 8 in; (**a**) *mdx* mouse myoblasts transfected with the exon 7 and 5 PMOs (20, 10, 5, 2.5, and 1 µM) and compared to untreated (UT) myoblasts; (**b**) *mdx* mouse myoblasts transfected the exon 7 Smn-PPMO (2, 1, 0.5, 0.25 and 0.1 µM) and compared to untreated (UT) myoblasts, with the graphed densitometry of (**a**) and (**b**) shown in (**c**) and (**d**) tissue harvested from *mdx* mice treated with either an un-conjugated peptide, unconjugated PMO (3x subcutaneous (2 nmol) injections) or the Smn-PPMO (1x subcutaneous (2 nmol) and 1x intraperitoneal (2 nmol) injections), with the graphed densitometry shown in (**e**). Tissues evaluated include the tibialis anterior (T), diaphragm (D), heart (H), kidney (K) and liver (L). The full length transcript (FL, 430 bp) and exon skipped products (delta-7(Δ7), 380 bp) are indicated by the lines to the left of the gel. The percentage of Δ7-*Smn* transcripts, as determined by densitometry, are shown below each lane. A 100 bp size marker was used for size comparison and a no template RT-PCR control (NTC) was loaded in the final lane.

The best exon 7 and exon 5 skipping AOs targeting the mouse sequence (m4 and m10) were synthesized as unmodified PMOs and transfected uncomplexed at 20, 10, 5, 2.5 and 1 µM into *mdx* myoblasts, and the transfected cells incubated for 3 days. Interestingly, RT-PCR results (Figure 5a) show the PMOs to be equally effective at inducing skipping of their target exons, with a maximum of 54–55% exon skipping achieved in myoblasts transfected with either PMO. Looking at the levels of exon skipping at the low 2.5 and 1 µM doses, it appears that the PMO targeting exon 7 may be marginally more effective than the PMO targeting exon 5, inconsistent with the earlier results obtained when testing 2′-*O*-methyl PS AOs.

Given that the exon 7 skipping PMO was most effective, and that 25 bases is preferred to reduce the cost and maintain ease of PMO synthesis, the **m3** sequence, targeting m7A(+ 07 + 31), was chosen as a positive control to take forward for in vitro evaluation as a PPMO. The Smn-PPMO was transfected into *mdx* myoblasts (2, 1, 0.5, 0.25 and 0.1 µM), and the transfected cells incubated for 3 days prior to *Smn* analysis (Figure 5b). Although increased levels of exon 7 skipping were observed using these concentrations in human cells, the mouse-specific Smn-PPMO only induced a maximum of 45% exon 7 skipping in mouse cells. This finding was consistent with the results obtained when testing 2′-*O*-methyl PS AOs in mouse cells, further supporting the concept that mouse-derived muscle cells may have a threshold of 50% exon 7 skipping.

To further demonstrate the advantage of a positive control AO sequence, the mouse-specific PPMO was evaluated in vivo as proof-of-concept for tissue delivery following chemistry optimization. *Mdx* mouse pups (postnatal day 5) were administered the Smn-PPMO by subcutaneous (2 nmol) and intraperitoneal (2 nmol) administration. The peptide-only control and the Smn-PMO were included as controls and administered to *mdx* mouse pups three times subcutaneously (2 nmol), with each injection administered at different sites (flanks and scruff) over five days. Seven days after the initial treatment, mice were sacrificed and tibialis anterior muscle (T), diaphragm (D), heart (H), kidney (K) and liver (L) were sampled for *Smn* analysis (Figure 5d). While no *Smn* exon 7 skipping was detected in mice treated with the uncomplexed PMO or the peptide alone, clear exon 7 skipping was evident in all tissues evaluated from the Smn-PPMO-treated mice, including a hint of exon skipping (8%) observed in the myocardium, a notoriously difficult-to-transfect tissue [36,37].

## 4. Discussion

The number and diversity of antisense applications has greatly expanded over the past decade, and as a result, so too has the art of AO design and optimization. Refinements and modifications to AO chemistries and subsequent methods for enhancing delivery continue to evolve. For efficient optimization of AO chemistry and delivery, we suggest that a positive control AO, targeting a ubiquitously expressed gene transcript, should be used during novel AO evaluation. Here, we report AO sequences targeting the human and mouse *SMN*/*Smn* genes that could be utilized in both in vitro and in vivo models to assess AO delivery.

Antisense oligomers were initially designed to induce skipping of the alternatively spliced exons 5 and 7 from *SMN1*/*SMN2* in normal human fibroblasts. Antisense oligomers designed to target across exon 7 were very effective at inducing exon skipping, with over 90% skipping of the target exon induced in cells transfected with 100 nM of the AO. The most efficient AO sequences, H7A(+ 07 + 31) and H7A(+ 13 + 32), anneal over an exonic splicing enhancer site, predicted to recruit binding of a number of positive splicing factors, predominately, Tra2β. These AOs were both effective at inducing almost complete exon skipping and could be used interchangeably when different AO sequence lengths are preferred.

Interestingly, while *SMN* exon 5 had a weaker predicted splice site score when compared to exon 7 (Table 3), efficient exon 5 skipping was not achieved by either AO transfected in human fibroblasts. Of particular note, one exon 5 targeting AO, H5A(+ 29 + 53), induced up to 100% transcript knockdown, with a similar result observed by the homologous mouse-specific AO targeting the same region, M5A(+ 32 + 56). These AOs target a somewhat repetitive region and have a high guanine (G) content (17 out of 25 bases), and as such have very high melting temperatures of 87 ᵒC and 86 ᵒC, respectively. High G-content AOs with contiguous stretches of Gs are reported to induce G-quartets and unique secondary structures that can have a multitude of effects on cell biology, including interaction with transcription inhibitors [38]. Taken together, these factors may further exacerbate the reported off-target binding effects of negatively charged phosphorothioate AOs, which we have previously shown to influence *SMN* transcript levels [14]. Intriguingly, AOs with a higher G-content were observed in this prior study to induce off-target protein accumulation within the cytoplasm. Indeed, these sequences are very G-rich, and are challenging for PMO synthesis with many commercial suppliers reluctant to make these compounds. Given that the results of exon 7 skipping were so impressive, we chose not to further pursue AOs to induce exon 5 skipping from the human gene transcript.

As a proof-of-concept for optimizing AO chemistry and delivery into multiple cell lines, the lead human *SMN* exon 7 skipping AO sequence was synthesized as a PMO, and a PMO conjugated to a PepK cell penetrating peptide tag to enhance delivery (PPMO) [32]. As expected, PepK dramatically improved cellular uptake and AO-induced *SMN* splicing efficiency when evaluated in normal human fibroblasts. Antisense oligomer delivery into fibroblasts is well established and routinely performed in our laboratory; however, AO delivery into neuronal cells is less efficient and requires an assisted delivery, such as PepK-mediated enhanced cellular uptake. To establish the effective concentration required for delivery into neuronal cells, the SMN-PPMO was evaluated in differentiated SH-SY5Y cells. The SMN-PPMO was highly effective at inducing *SMN* exon 7 skipping at the higher concentrations, confirming the use of the AO sequence as a positive control for neuronal cells.

To identify a positive control AO for use in mouse cells and for in vivo studies, mouse *Smn*-specific sequences were designed and transfected into *mdx* myogenic cells. Interestingly, mouse-specific PMOs were only able to induce up to 45–60% exon 7 skipping, compared to the 100% targeted skipping achieved in primary human fibroblasts. Differences in the predictive strength of the exon 7 splice site scores between the species did not meet the threshold expected to alter splicing outcomes and may not fully account for the differences in exon skipping efficiency. Transcripts from both the *SMN1* and *SMN2* genes are amplified from the human RNA, and, therefore, the endogenous excision of exon 7 that is the result of poorer exon 7 selection in the *SMN2* gene transcripts, unique to human cells and not present in mice, could in part explain the difference in exon 7 skipping efficiency between species.

Differences in splicing factors and splicing regulation between species may also contribute to differences in AO-mediated exon-skipping effectiveness. We have previously reported these differences in AO targeting of the human and mouse dystrophin gene transcripts [26]. Dystrophin transcript exon skipping studies have shown that certain exons are more difficult to skip than others, with some requiring combinations of AOs to achieve efficient exon skipping [17]. Transfection of cocktails of AOs targeting mouse *Smn* exon 7 marginally improved exon skipping levels when compared to transfection of individual AOs, but did not justify the ongoing use of AO combinations. It appears that only a maximum of 60% AO-induced skipping of mouse *Smn* exon 7 can be achieved, compared to the near 100% skipping induced in the human *SMN* transcripts. Regardless of how highly conserved the *SMN* and *DMD* transcripts are between humans and mice, taken together, these results emphasize the disparities in splicing between the species, possibly reflecting the context of the exon and the influence of intron structure and therefore, the need for species-specific control AO sequences. This finding is also important for the design of therapeutic AOs, whereby species-specific AO sequences should be designed to target across the exon and be screened prior to in vivo application.

In comparison to 2′-*O*-methyl AOs targeting *Smn* exon 7, 2′-*O*-methyl, AOs targeting *Smn* exon 5 induced up to 100% exon 5 skipping in *mdx* myoblasts, compared to 50% exon 7 skipping, suggesting that exon 7 splicing is more tightly regulated than exon 5 in the mouse transcript. This finding was supported by the predicted splice site MES scores, suggesting *Smn* exon 5 to be a less efficiently spliced exon in the mouse model compared to exon 7. Interestingly, when the sequences were synthesized as PMOs, there was no obvious difference in the levels of skipping of the different target exons. We therefore recommend that AOs targeting *Smn* exon 5 and 7 could be used interchangeably as positive control AOs. However, there may be chemistry-dependent differences in AO-mediated skipping efficiency; indeed, it is not recommended that AOs with such a high CG content be synthesized as PMOs, as this can lead to poor water solubility and may account for reduced antisense activity of the exon 5 PMO [39].

The use of a ubiquitously expressed positive control AO sequence is of great importance when optimizing AO sequence, chemistry, delivery and tissue targeting for in vivo studies. Here, we provide evidence for the use of the *Smn* exon 7 skipping AO as an ideal control for assessing tissue delivery following systemic AO administration of two AO chemistries: the PMO and the PepK-conjugated PPMO. Consistent with our in vitro data, use of the PepK conjugate markedly improved cellular uptake and antisense effects. While mice treated with the unconjugated Smn-PMO showed no evidence of exon 7 skipping in the tissues evaluated, mice treated with the peptide-conjugated Smn-PPMO clearly demonstrated exon 7 skipping within each of the tissues. The use of the positive control PMO permitted exon 7 skipping to be quantified and assessed delivery to specific tissue types prior to evaluation of therapeutic antisense molecules in future studies. This has huge benefits for the development and evaluation of novel AO chemistries or cell penetrating peptides designed for specific cellular delivery and uptake. Given the ubiquitous expression of *Smn*, this AO sequence can be utilized to assess tissue delivery of a multitude of AO chemistries via all routes of administration, including those specifically targeting the central nervous system.

## 5. Conclusions

We report positive control AOs targeting the ubiquitously expressed *SMN* genes, that can be used as transfection controls and for optimization of both in vitro and in vivo experiments. These sequences could be synthesized using any current or future chemistry suitable for steric-blocking applications. Following AO sequence screening and evaluation of multiple AO chemistries, we recommend the following sequences (Table 4) as positive control AOs for both human and mouse transfections and for in vivo evaluation:

## Figures and Tables

**Table 1:** Sequences and binding coordinates of exon skipping AOs designed for the human *SMN* and mouse *Smn* transcripts. The AO length and melting temperature (Tm) of the 2′-*O*-methyl AO sequence is listed.

Species and Exon	AO Name/Number	Coordinates	Sequence 5′-3′	Length (bp)	Tm (ᵒC)
Human Exon 7	h1 h2 h3 h4 h5 h6 h7	SMN H7A(− 08 + 17) SMN H7A(+ 01 + 25) ^1^ SMN H7A(+ 07 + 31) ^1^ SMN H7A(+ 06 + 27) ^1^ SMN H7A(+ 13 + 32) ^1^ SMN H7A(+ 22 + 46) ^1^ SMN H7D(+ 17 − 13)	UGA UUU UGU CUA AAA CCC UGU AAG G CUU CUU UUU GAU UUU GUC UAA AAC C ACC UUC CUU CUU UUU GAU UUU GUC U UCC UUC UUU UUG AUU UUG UCU G CAC CUU CCU UCU UUU UGA UU UUA AGG AAU GUG AGC ACC UUC CUU C CUG GCA GAC UUA CUC CUU AAU UUA AGG AAU	25 25 25 22 20 25 30	66.4 58.6 64.7 59.5 59.2 70.5 70.2
Human Exon 5	h8 h9	SMN H5A(+ 04 + 28) SMN H5A(+ 29 + 53)	GUG GUG GGC CAU UGA AUU UUA GAC C UGG GGU GGU GGU GGU GGC GGU GGC G	25 25	71.1 87.0
Mouse Exon 7	m1 m2 m3 m4 m5 m6 m7 m8	Smn M7A(+ 02 + 31) Smn M7A(+ 06 + 27) Smn M7A(+ 07 + 31) Smn M7A(+ 07 + 36) ^2^ Smn M7A(+ 13 + 32) Smn M7D(+ 11 − 14) Smn M7D(+ 11 − 19) Smn M7D(+ 17 − 13)	ACU UUC CUU CUU UUU UAU UUU GUC UGA AAC UCC UUC UUU UUU AUU UUG UCU G ACU UUC CUU CUU UUU UAU UUU GUC U UGA GCA CUU UCC UUC UUU UUU AUU UUG UCU CAC UUU CCU UCU UUU UUA UU AAU GAC AGA CUU ACU UCU UAA UUU G UUU AAA AUG ACA GAC UUA CUU CUU AAU UUG AUG ACA GAC UUA CUU CUU AAU UUG UAU GUG	30 22 25 30 20 25 30 30	63.5 56.9 59.8 67.2 52.6 60.7 61.9 65.9
Mouse Exon 5	m9 m10 m11 m12 m13	Smn M5A(− 06 + 19) Smn M5A(+ 12 + 36) Smn M5A(+ 32 + 54) Smn M5A(+ 56 + 77) Smn M5A(+ 18 − 07)	CGU UGA AUU UUA GAC CUG GCU AUA A AGG CGG CGG CGG CGG GCC GUU GAA U AAG GGG GGA GGG GGU AGU GGA GGC G AAC GGG GGC AUC CAG CAC GGC AG UAC UUA CUG GUG GUC CUG AAG GGA A	25 25 25 23 25	65.8 86.8 85.9 81.6 75.7

^1^ Some sequence overlap with 2′-*O*-methoxyethyl AOs described in [25]. ^2^ Previously used as a control [4].

**Table 2:** PCR conditions for human *SMN* and mouse *Smn* transcript amplification.

Transcript Sizes	Primer	Primer Sequence	Temperature Profile
*SMN* FL 404 bp Δ5 308 bp Δ7 350 bp	Exon 4 Fwd Exon 8 Rev	AGGTCTCCTGGAAATAAATCAG TGGTGTCATTTAGTGCTGCTCT	55 °C 30 min 94 °C 2 min 25 cycles: 94 °C 40 s 56 °C 1 min 68 °C 1 min
*Smn* FL 430 bp Δ5 334 bp Δ7 380 bp	Exon 4 Fwd Exon 8 Rev	GAAAGTCAAGTTTCCACAGACG CACCCCATCTCCTGAGACAGAGC	55 °C 30 min 94 °C 2 min 28 cycles: 94 °C 40 s 60 °C 1 min 68 °C 1 min

**Table 3:** Predicted splice site scores of human *SMN1* and mouse *Smn* exons 5 and 7 using MaxEntScan. Nucleotides in CAPITALS indicate exon sequence, lower case nucleotides represent intronic sequences.

Species	Exon	Splice Site	Splice Site Sequence	MES Score
Human	5	Acceptor Donor	ctttgaaatattccttatag CCA CCAgtaagt	5.44 9.09
	7	Acceptor Donor	ttcctttattttccttacagGGT GGAgtaagt	10.92 8.57
Mouse	5	Acceptor Donor	cttggaaatattctttatagCCA CCAgtaagt	3.29 9.09
	7	Acceptor Donor	tttatatgctctctttacagGGT GAAgtaagt	11.13 9.82

**Table 4:** Sequences and binding co-ordinates of recommended positive control exon skipping AOs designed for the human *SMN* and mouse *Smn* transcripts.

Species	Coordinates	Sequence 5′-3′
Human	SMN H7A(+ 07 + 31) SMN H7A(+ 13 + 32)	ACC UUC CUU CUU UUU GAU UUU GUC U CAC CUU CCU UCU UUU UGA UU
Mouse	Smn M7A(+ 07 + 31) Smn M5A(+ 12 + 36)	ACU UUC CUU CUU UUU UAU UUU GUC U AGG CGG CGG CGG CGG GCC GUU GAA U

## Data Availability

Not applicable.

## Supplementary Materials

The following are available online at https://www.mdpi.com/article/10.3390/biomedicines9050552/s1, Figure S1: Analysis of cocktails of 2′-*O*-methyl AOs targeting mouse *Smn* exon 7.

## Abbreviations

AO	antisense oligonucleotide
SMN	survival motor neuron
SMA	spinal muscular atrophy
PMO	phosphorodiamidate morpholino oligomer
PPMO	peptide-conjugated phosphorodiamidate morpholino oligomer
