# Supplementary material for: Targeted SMN Exon Skipping: A Useful Control to Assess In Vitro and In Vivo Splice-Switching Studies
Source: Biomedicines. 2021 May 14;9(5):552. doi: 10.3390/biomedicines9050552 (PMC8156830; doi:10.3390/biomedicines9050552)
Supplement: Supplementary file 1 [file biomedicines-09-00552-s001.zip › biomedicines-1205425-supplementary.pdf]

biomedicines

MDPI

Review

From Antisense RNA to RNA Modification: Therapeutic Potential of RNA-Based Technologies

Hironori Adachi [1], Martin Hengesbach [2], Yi-Tao Yu [1,*] and Pedro Morais [3,*]

Supplementary figure

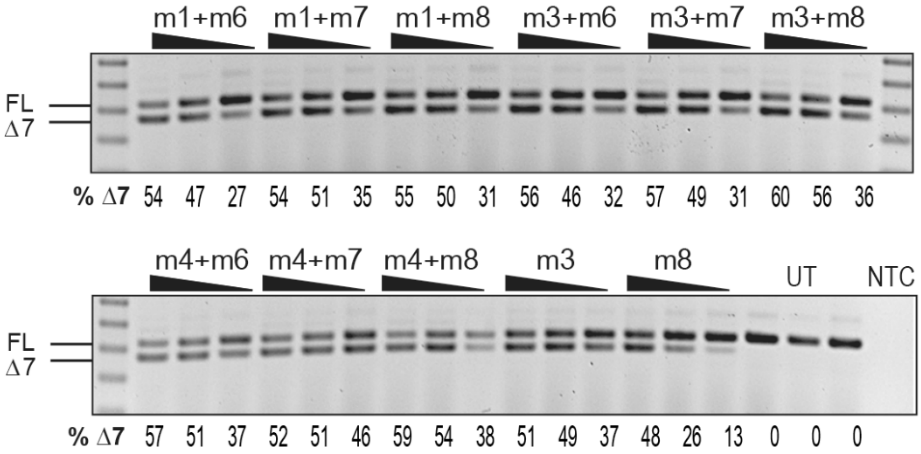

Figure S1. RT-PCR analysis of *Smn* transcripts across exons 4 to 8 in *mdx* mouse myoblasts transfected with combinations of 2´OMethyl PS AOs at total concentrations of 200, 100 and 50 nM.
